# Acute Lumbar Pain Revealing a Rare Localization of a Primary Squamous Cell Carcinoma of the Psoas

**DOI:** 10.7759/cureus.102654

**Published:** 2026-01-30

**Authors:** Achraf Benamou

**Affiliations:** 1 Urology, Mohammed VI University Hospital, Oujda, MAR

**Keywords:** brodie's abscess, psoas, psoas abscess, pyonephrosis, skeletal muscle metastasis, squamous cell carcinoma

## Abstract

Damages to the psoas muscle are most often of infectious origin; other causes are observed, notably neoplasia and metastasis, hematomas, which sometimes pose a problem of care, especially in tumor-related conditions. Here, we report a rare case of psoas abscess associated with left pyonephrosis, revealing a squamous cell carcinoma of the left psoas without other tumor sites, along with the diagnostic and therapeutic approach taken in this case. This case report will make clinicians think about psoas muscle lesions, especially tumor lesions, which require a well-defined therapeutic approach.

## Introduction

Lesions of the psoas muscle represent a heterogeneous group of conditions that include infectious, inflammatory, traumatic, and neoplastic etiologies. Among these, psoas abscesses are the most frequently encountered in clinical practice and may be classified as primary or secondary, the latter usually resulting from contiguous spread of infection from urological, gastrointestinal, or osteoarticular sources [1,2]. In contrast, tumoral involvement of the psoas muscle, whether primary or metastatic, remains rare and poorly recognized, often leading to delayed diagnosis.

Skeletal muscle metastases are an uncommon clinical entity despite the rich vascular supply and substantial mass of skeletal muscle tissue. Their reported incidence ranges from 0.8% to 16% in autopsy series, reflecting significant underdiagnosis during life [3,4]. Several mechanisms have been proposed to explain this rarity, including continuous muscle contraction, high tissue pressure, lactic acid production, and a locally hostile immune environment that may inhibit tumor cell implantation and growth [5].

When present, skeletal muscle metastases pose major diagnostic challenges. Clinical manifestations are often nonspecific, including pain, swelling, fever, or functional impairment, and may closely mimic infectious processes, such as abscesses or inflammatory myositis [6]. Radiological findings are similarly nonspecific, particularly in the psoas muscle, where abscess, hematoma, and tumor can share overlapping imaging features. These difficulties are further compounded by the coexistence of superinfection or adjacent osseous involvement, which may obscure the underlying malignant nature of the lesion [7].

We report a rare and diagnostically challenging case of a left psoas abscess associated with left pyonephrosis, ultimately revealing a squamous cell carcinoma of the psoas muscle without identification of a primary tumor. This case underscores the importance of considering neoplastic etiologies in atypical psoas lesions and highlights the role of histopathological confirmation in guiding management.

## Case presentation

Patient information

A 68-year-old patient with no significant medical or toxicological history presented with symptoms dating back one month before hospitalization. He reported left lower back pain, fever, deterioration of general condition, and unquantified weight loss.

Clinical findings and diagnostic assessment

Physical examination revealed marked left lumbar tenderness. Initial laboratory evaluation showed an inflammatory syndrome with hyperleukocytosis (18,000/mm^3^), elevated C-reactive protein (CRP: 252 mg/L), and a sterile urine culture (Table 1).

**Table 1:** Table summarizing the results of the biological examination.

Biological parameters	Result	Reference range (indicative)	Interpretation
White blood cells (WBC)	18,000/mm^3^	4,000-10,000/mm^3^	Hyperleukocytosis
C-reactive protein (CRP)	252 mg/L	<5 mg/L	Significant inflammatory syndrome
Urine culture	Sterile	Sterile	No urinary tract infection

**Figure 1:**
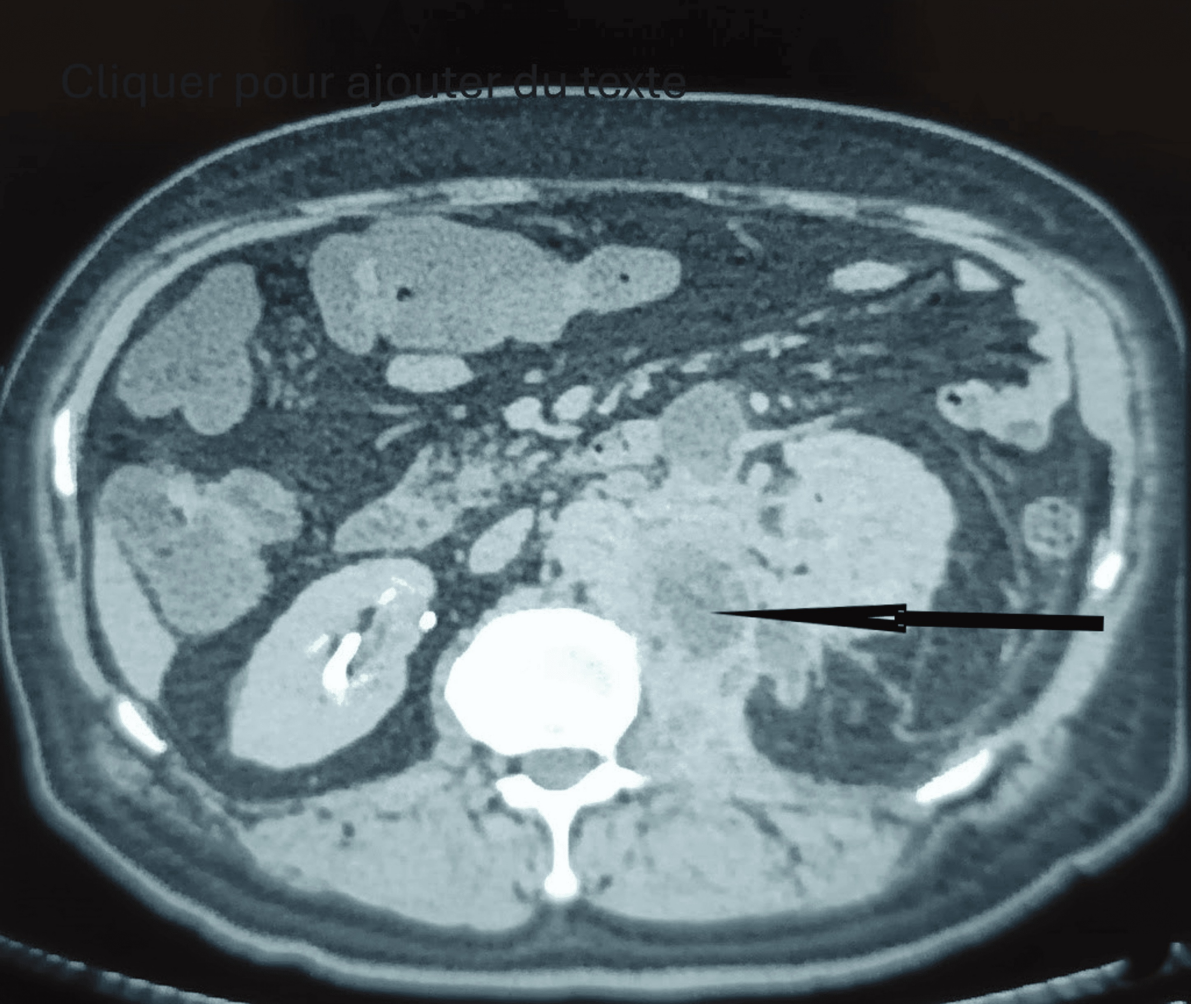
CT scan shows the infiltration of the lumbar portion of the left psoas muscle, which appears enlarged and heterogeneous, containing multiple fluid collections (arrow).

A CT scan demonstrated marked infiltration of the lumbar portion of the left psoas muscle, which appeared enlarged and heterogeneous, containing multiple fluid collections, the largest measuring 41 mm. Associated findings included left pyonephrosis, thrombosis of the left renal vein, and erosion of the lateral aspect of the T12 vertebral body (Figure 1).

**Figure 2:**
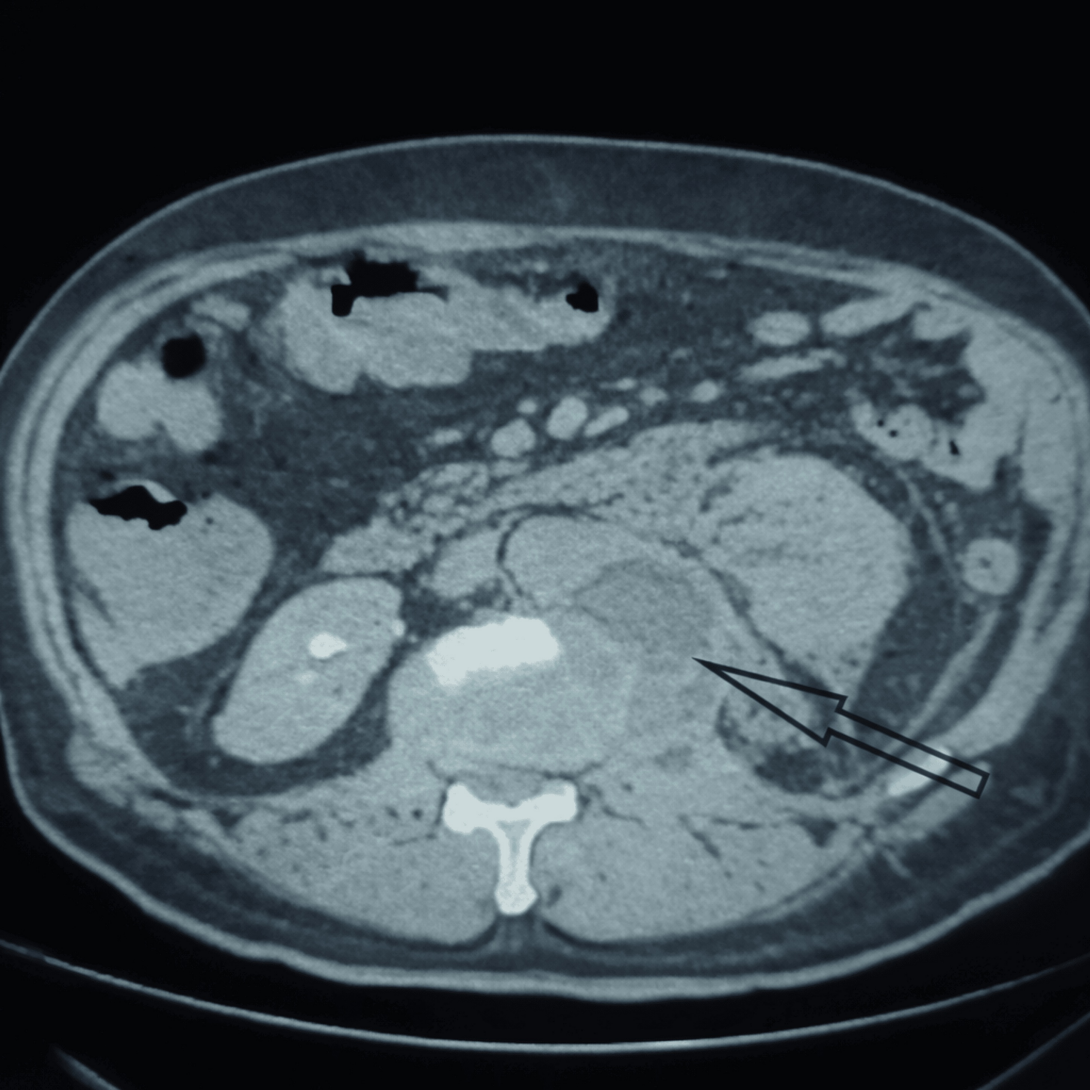
Lytic lesion with cortical destruction at T12, the existence of a tumor process should not be excluded.

Given this clinical and paraclinical presentation, several diagnostic hypotheses were considered, primarily renal tumor and urogenital tuberculosis. Additional investigations were performed accordingly. A follow-up CT scan was obtained to further characterize the psoas lesion and to reassess after antibiotic therapy, particularly to pursue the etiological diagnosis. It revealed a large collection measuring 16 cm at the lumbar head of the left psoas muscle, infiltrating the medial border of the left kidney, persistent thrombosis of the left renal vein, and a lytic lesion with cortical destruction at T12, which was inconclusive, failing to differentiate clearly between an infectious or tumoral process (Figure 2).

**Figure 3:**
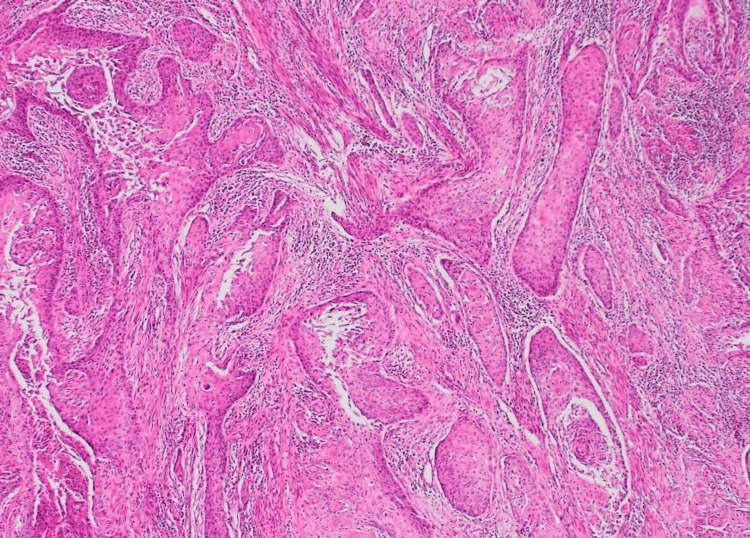
Histological section shows nests of atypical epithelial cells with eosinophilic cytoplasm and central keratin pearls infiltrating a chronically inflamed stroma, diagnostic of a well-differentiated squamous cell carcinoma.

**Figure 4:**
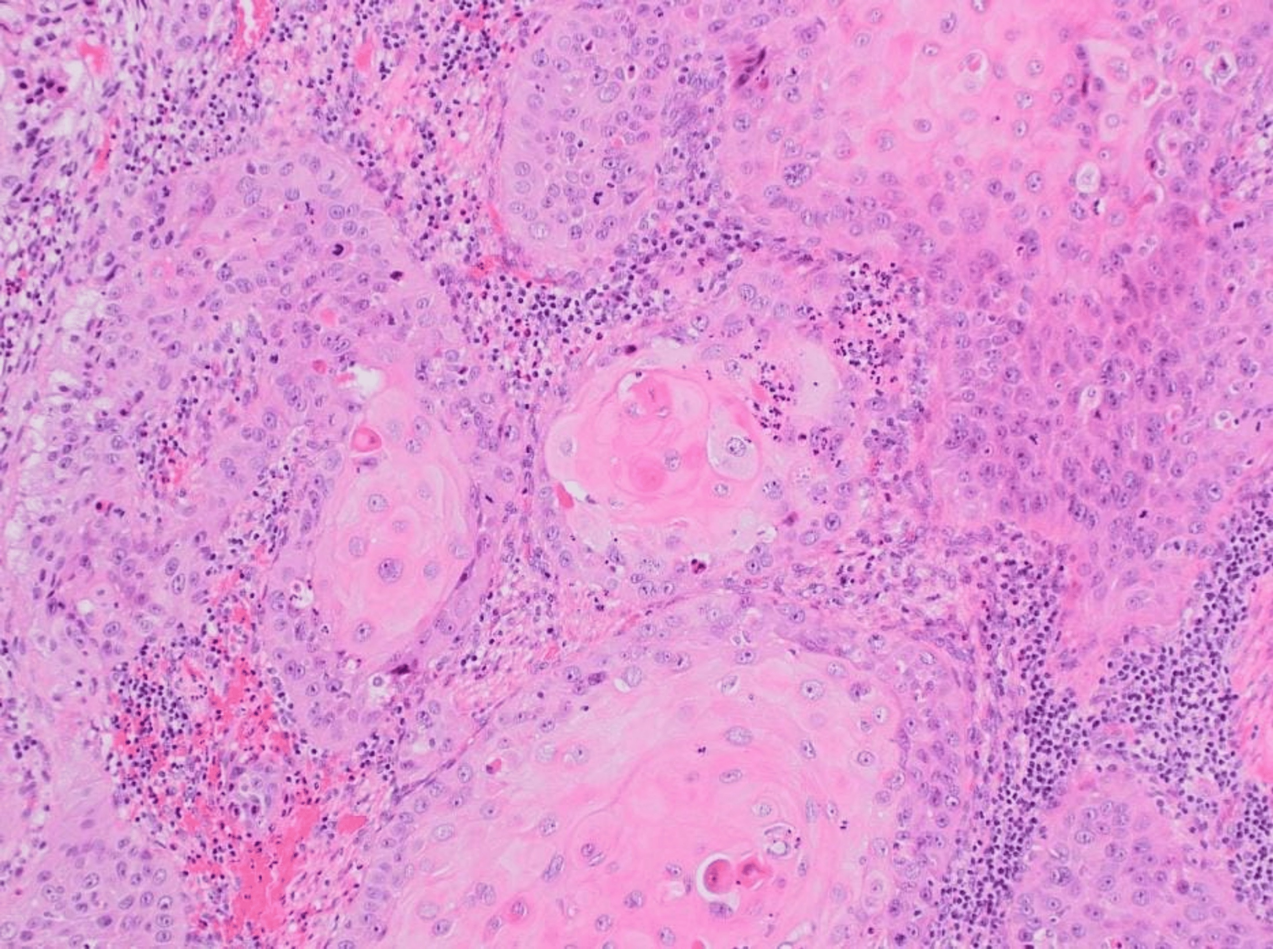
Histological image reveals nests of atypical epithelial cells infiltrating the stroma with characteristic keratin pearls, confirming a differentiated squamous cell carcinoma.

A chest CT scan showed a moderate left pleural effusion. Thoracentesis with pleural biopsy was performed to search for tuberculosis; testing for *Mycobacterium tuberculosis* in both pleural fluid and urine was negative. Given the persistence of diagnostic uncertainty, a CT-guided biopsy of the left psoas muscle was performed. Histopathological examination revealed epidermoid (squamous cell) carcinoma of the left psoas muscle (Figures 3, 4).

Therapeutic intervention and follow-up

The initial therapeutic strategy targeted a presumed infectious cause; the patient was started on triple-antibiotic therapy. Surgical or percutaneous drainage was postponed until a definitive diagnosis was established, particularly to exclude a tumor. This was ultimately confirmed by the biopsy showing squamous cell carcinoma of the psoas muscle without evidence of another primary site.

The patient was scheduled to receive external radiotherapy with cobalt-60 in combination with concomitant chemotherapy. However, the clinical course quickly worsened. He developed severe thromboembolic complications and refractory malignant hypercalcemia, leading to death.

Patient perspective

The patient expressed satisfaction with the care provided throughout the treatment course, prior to the rapid clinical deterioration related to thromboembolic complications and malignant hypercalcemia.

## Discussion

Psoas muscle lesions represent a diagnostic challenge due to the anatomical location of the muscle, its proximity to retroperitoneal organs, and the broad spectrum of potential etiologies. While infectious causes, particularly primary and secondary psoas abscesses, remain the most common, clinicians must remain aware of less frequent but clinically significant conditions such as neoplastic infiltration and metastatic disease [1,2]. The present case illustrates the complexity of diagnosing psoas pathology when infectious and malignant processes coexist.

Skeletal muscle metastases are rare events in oncological practice, a paradox given the high vascularity and volume of skeletal muscle tissue. Several hypotheses have been proposed to explain this rarity, including mechanical effects of constant muscle contraction, high tissue oxygenation, lactic acid accumulation, and locally enhanced immune surveillance that may inhibit tumor cell implantation and proliferation [3,5]. Despite these protective factors, metastases can occur, particularly in advanced disease or in aggressive tumor subtypes.

The iliopsoas muscle appears to be one of the preferential sites for skeletal muscle metastases, possibly due to its rich blood supply, extensive lymphatic drainage, and anatomical proximity to major vascular and visceral structures [4,6]. In most reported cases, muscle metastases are associated with disseminated disease, and the identification of an isolated psoas metastasis, especially in the absence of an identifiable primary tumor, remains exceptional. Squamous cell carcinoma presenting as a skeletal muscle metastasis is particularly rare and has been only sporadically reported in the literature [8].

Clinically, muscle metastases may present with a wide range of symptoms, from asymptomatic masses to severe pain, functional impairment, and systemic manifestations such as fever and weight loss. When tumor necrosis or secondary infection occurs, the clinical picture may closely resemble that of an abscess, leading to misdiagnosis and delayed oncological management [6]. In the present case, the superinfection of the psoas lesion and the associated pyonephrosis were the initial clinical manifestations, effectively masking the underlying malignant process.

Radiological evaluation is essential but frequently inconclusive. Computed tomography is typically the first-line imaging modality and may reveal an enlarged muscle with a hypodense lesion, central necrosis, and peripheral enhancement following contrast administration. These findings are highly suggestive of an abscess but are not specific and may also be seen in hemorrhage or necrotic tumors [5,7]. Magnetic resonance imaging provides superior soft tissue contrast and can better delineate the extent of muscle and adjacent osseous involvement; however, signal characteristics often overlap between infectious and neoplastic lesions, limiting its diagnostic specificity [9]. Consequently, imaging alone is insufficient to establish a definitive diagnosis in many cases.

In this context, image-guided percutaneous biopsy plays a pivotal role and should be considered early when clinical evolution or imaging findings are atypical or when response to antibiotic therapy is suboptimal. Histopathological examination remains the gold standard for diagnosis and is essential for guiding further management. In our patient, biopsy of the psoas muscle was decisive in revealing a squamous cell carcinoma and allowed exclusion of other differential diagnoses, such as tuberculosis or primary renal malignancy.

From a therapeutic standpoint, the management of skeletal muscle metastases is not standardized and largely depends on tumor histology, extent of disease, and the patient’s general condition. Treatment is most often palliative, aiming to control symptoms and improve quality of life. Available options include radiotherapy, systemic chemotherapy, and, in selected cases with isolated or symptomatic lesions, surgical resection [3,8]. In our case, despite planning combined radiochemotherapy, the rapid clinical deterioration highlighted the aggressive nature of the disease and the poor prognosis associated with muscle metastases.

Overall, this case emphasizes the importance of a multidisciplinary approach involving urologists, oncologists, radiologists, pathologists, and infectious disease specialists. Early consideration of neoplastic etiologies in atypical or refractory psoas lesions may help avoid diagnostic delays, prevent inappropriate interventions, and facilitate timely initiation of appropriate oncological treatment.

## Conclusions

Skeletal muscle metastases are rare and often represent a diagnostic challenge because of their nonspecific clinical and radiological presentation, particularly when associated with superinfection or adjacent osseous involvement. These atypical presentations may delay diagnosis and contribute to inappropriate initial management. A high index of suspicion is therefore essential when evaluating unusual muscular lesions, even in the absence of an identified primary malignancy.

Management requires a multidisciplinary approach and is generally palliative, guided by the patient’s overall condition, tumor biology, and extent of metastatic disease. Although prognosis remains poor, early recognition and accurate diagnosis of skeletal muscle metastases may allow timely initiation of appropriate therapy, optimize symptom control, and avoid unnecessary diagnostic or therapeutic delays.
